# Communication training for advanced medical students improves information recall of medical laypersons in simulated informed consent talks – a randomized controlled trial

**DOI:** 10.1186/1472-6920-13-15

**Published:** 2013-02-01

**Authors:** Anne Werner, Friederike Holderried, Norbert Schäffeler, Peter Weyrich, Reimer Riessen, Stephan Zipfel, Nora Celebi

**Affiliations:** 1 Department for Internal Medicine VI, Psychosomatic Medicine, University Hospital of Tübingen, Osianderstr. 5, 72076, Tübingen, Germany; 2 Office of the Dean of student affairs, Medical Faculty, University of Tübingen, Geisweg 5, 72076, Tübingen, Germany; 3 Department for Internal Medicine IV, Endocrinology, Diabetology, Nephrology, Angiology and Clinical Chemistry, University Hospital of Tübingen, Otfried-Müller-Str. 10, 72076, Tübingen, Germany; 4 Medical Intensive Care Unit, University Hospital of Tübingen, Otfried-Müller-Str. 10, 72076, Tübingen, Germany

**Keywords:** Medical student, Medical layperson, Informed consent talk, Communication training, Cognitive load

## Abstract

**Background:**

Informed consent talks are mandatory before invasive interventions. However, the patients’ information recall has been shown to be rather poor. We investigated, whether medical laypersons recalled more information items from a simulated informed consent talk after advanced medical students participated in a communication training aiming to reduce a layperson’s cognitive load.

**Methods:**

Using a randomized, controlled, prospective cross-over-design, 30 5th and 6th year medical students were randomized into two groups. One group received communication training, followed by a comparison intervention (early intervention group, EI); the other group first received the comparison intervention and then communication training (late intervention group, LI). Before and after the interventions, the 30 medical students performed simulated informed consent talks with 30 blinded medical laypersons using a standardized set of information. We then recorded the number of information items the medical laypersons recalled.

**Results:**

After the communication training both groups of medical laypersons recalled significantly more information items (EI: 41 ± 9% vs. 23 ± 9%, p < .0001, LI 49 ± 10% vs. 35 ± 6%, p < .0001). After the comparison intervention the improvement was modest and significant only in the LI (EI: 42 ± 9% vs. 40 ± 9%, p = .41, LI 35 ± 6% vs. 29 ± 9%, p = .016).

**Conclusion:**

Short communication training for advanced medical students improves information recall of medical laypersons in simulated informed consent talks.

## Background

Pre-interventional informed consent talks (ICT) are a crucial component in patient management. They are mandatory for most invasive therapeutic and diagnostic procedures. The elements of adequate informed consent include disclosure, understanding, decision-making capacity, and voluntary participation [1]. Technically, every intervention constitutes bodily harm and requires the patient’s permission (“informed consent”) [2]. Informed consent aims at enabling patients to make autonomous decisions about a medical procedure and prevent potential harm, though in clinical practice, the main emphasis is on the doctors’ legal protection rather than ensuring the patient’s understanding [3,4]. A good understanding of the planned procedure with all its implications is needed in order to enable the patient to give consent in an informed way [2]. However, several studies have shown a poor recall of facts conveyed during the informed consent talks, especially in medical laypersons [5-7]. Furthermore, comprehension of risks and benefits has been shown to be relatively poor – in research conditions as well as clinical settings - and there is a clear discrepancy between the patients’ perception of their level of understanding and the actual knowledge they show when tested [8-10]. Laypersons have a particular difficulty estimating the single event probability in relation to their own case [11].

A substantial amount of research has been done on informed consent in clinical trials [6,12]. A systematic review by Flory and colleagues showed that most studies focused on factors concerning the patients, like age and literacy. A lower educational background was clearly associated with less comprehension; gender had no influence on the ability to understand; and age had inconsistent effects, with some studies pointing toward a reduction of understanding in patients older than 50 [13]. Surprisingly little has been written about interventions that improve patients’ understanding (e.g. additional audiotapes, a summarizing letter, question prompt sheets) and the results are not consistent according to a review by van der Meulen et al. [14]. Investigations concerning communication training for doctors conducting the ICTs are scarce. Brown and colleagues designed a communication training for oncologists seeking informed consent for clinical trials, which had a limited success since the patients only showed more positive attitude but did not recall more information from the talks [15].

To our knowledge, factors regarding the person conducting the ICT have been mostly neglected. This seems surprising, as another explanation for poor recall in patients could be the fact that doctors are poorly trained to perform these talks. Nückles et al. used information technology specialists to show that sensitization of experts to the layperson’s knowledge-level improves the efficacy of their communication [16]. It is well known that patients’ reactions are linked to doctors’ communication strategies. Patients were more likely to join clinical trials, if their doctor described the study benefits and side effects properly [17]. Strategies to improve talks have been developed in other fields of medicine, where precise information delivery is crucial (i.e. breaking bad news) [18]. Appropriate ICT training is not always available. Furthermore, it is still common practice to require newly graduated junior doctors, who lack experience in explaining the procedure, to conduct ICTs [19].

From a didactical point of view, an ICT with a medical layperson is a short, very dense lecture regarding an unfamiliar topic that usually has to be learned in stressful circumstances. As literature shows, understanding of complex tasks can be improved by using certain techniques that reduce the cognitive load [20].

According to the cognitive load theory, new information is processed in the working memory, which has a limited capacity. It can hold up to 5–9 new pieces of information for about 20 seconds. Thus, cognitive overload prevents learning [20]. Cognitive load comprises three components:

i) Intrinsic cognitive load, which is mainly determined by the complexity of the topic to be learned and the interactivity of its information items,

ii) The extrinsic cognitive load, which is determined by the way information is presented, and

iii) The germane cognitive load, which is the effort involved in the actual learning process [20].

The intrinsic cognitive load in the ICTs is usually determined by the procedure and therefore a reduction in the intrinsic cognitive load without jeopardizing the information content of the ICT can only be done to a limited extent. The main effort should be focused on optimization of the extrinsic cognitive load with indirect reduction of the intrinsic cognitive load, and simultaneous preservation of the actual content.

Based on these principles, we conducted a randomized controlled cross-over study. We investigated, whether a short communication training that focused on teaching advanced medical students how to reduce a layperson’s cognitive load improves a medical laypersons’ information recall in a simulated informed consent talk.

## Methods

### Pilot study

We conducted a pilot study with 9 final-year medical students in order to estimate the sample size. The appropriate sample size was calculated using nQuery 7.0 (Statistical Solutions, Saugus, MA, U.S.A.). A sample size of at least 12 students in each group would allow the desired detection of 15% improvement in number of remembered information items (three or more) with a power of 0.8 and a significance level of 0.05.

### Study design

We conducted a prospective randomized controlled cross-over study.

A total of 30 advanced medical students (5th and 6th year) with limited experience and no specific training in informed consent talks (ICT) were asked to perform ICTs with 30 volunteer healthy medical laypersons.

The students were randomized into two equally large groups using a table with random numbers. One group (early intervention, EI) received a communication training first and then a comparison intervention on ECG interpretation. The second group (late intervention, LI) first received the comparison intervention and then the communication training. The medical students, medical laypersons and the three different cases for the ICT were arranged so that every student met with three different medical laypersons, explaining each case once. The study was conducted as follows:

- On the day of the study, the medical students received a brief introduction to the study without disclosure of the study question. Meanwhile, the medical laypersons were instructed on their role during the encounter.

- The medical students conducted the first ICT (ICT_1_) without any further preparation. Each ICT lasted 15 minutes and the students were asked to convey a standardized set of 20 information items.

- After that, the medical students received their first training session, either the communication training or the comparison intervention, according to their randomization group. Both classes lasted 30 minutes and were taught by different instructors. Meanwhile the medical laypersons wrote down what they remembered from the first ICT (free text).

- Then the medical students conducted the second ICT (ICT_2_) that involved a different case and a new medical layperson,

- The next step was the second teaching session. The EI-group now had the class on ECG-interpretation, while the LI-group group received the communication training. Meanwhile the medical laypersons wrote down what they remembered from their second ICT.

- Finally, all students conducted their last ICT (ICT_3_) dealing with the third case and a third medical layperson. One more time, medical layperson wrote down everything they could remember immediately after the ICT.

**Figure 1:**
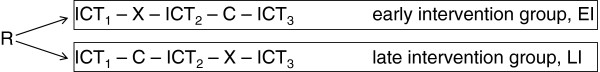
**Study design.** R: Randomization of 30 5th and 6th year medical students and 30 medical laypersons into early intervention group (EI) and late intervention group (LI). ICT: informed consent talk performed by an advanced medical student with a medical layperson. X: intervention (communication training for the medical students aiming to teach how to reduce the cognitive load). C: comparison intervention (lecture on ECG interpretation). Every medical student conducted three ICTs with three different medical laypersons.

The cases were permutated and served as first, second or third ICT equally often. Two blinded raters used a checklist to identify the standardized information. The study design is illustrated in Figure 1.

The medical students were blinded to the study question; the medical laypersons were blinded to both the study question and the intervention. The dependent variable was the number of information items written down by the medical laypersons. Based on consent of two experienced physicians, we considered the recollection of three additional details (15% of the information) as clinically relevant and thus as the smallest meaningful difference.

### Medical laypersons

The 30 medical laypersons were volunteers who received a small financial compensation. Inclusion criteria included an age between 35 and 70 years, reflecting the age group likely to undergo the procedures of the ICTs, and fluency in German. Volunteers were excluded if they or their close relatives suffered from major illness, had surgeries within the last 3 years, or had an occupation in the medical or medically related field (e.g. biology, neuroscience) in order to limit their level of medical knowledge. All volunteers received preparation materials before the study. As the content of our ICTs was not the medical condition itself, but the consequently necessary surgical procedure, the volunteers received some basic information about their alleged underlying diseases. Additionally, they received role instructions including a request to ask questions based on their understanding, but not to dominate the talks, as we wanted to allow students to structure their talks as learned and establish similar conditions for all students. They were randomly assigned to the randomized student groups and the order of the permutated cases.

### Medical students

A total of 120 5th and 6th year medical students were asked to participate in the study during a lecture. The students, who declined to participate in the study, were not required to provide a reason. The study was conducted during instruction-free time. The tudents gave written informed consent. All participating students had taken mandatory courses in communication, which included lectures and seminars on history taking and “breaking bad news”, role-plays and encounters with both standardized and real patients (a total of six hours). A total of 30 students agreed to participate and were randomized to either the early or the late intervention group (EI vs. LI). They, too, received a small financial compensation. One week before the study the medical students received written information about the study and were familiarized with the medical conditions used in the cases. They received modified versions of the informed consent forms commonly used in German hospitals with a standardized set of information items. All students were randomly assigned to the teaching sequence, the order of cases and patients to be encountered.

### Cases

We chose three different cases: severe aortic valve defect, kidney cancer, and pancreatic tumor. All three conditions were designed to be at a stage, which required a surgical intervention, which was supposed to be the content of the ICTs. We chose rather complex procedures in order to have 20 different standardized information items that had to be remembered per case. Two clinical experts confirmed that the chosen cases were appropriate for our study purpose. The cases were constructed in such a way that they all had the same amount of information items (n = 20), which we equaled to intrinsic cognitive load. These were grouped into standardized procedural steps for the operation itself (n = 5), the likelihood of success (n = 1), general (n = 5) and specific (n = 7) complications, and the necessary follow-up treatment (n = 2). An example is shown in Table 1.

**Table 1:** Example of a checklist

**Checklist aortic stenosis**	
**A.**	**Procedural steps intra-operatively**
A1.	Sternotomy
A2.	Opening of the pericardium
A3.	Connection to heart-lung-machine
A4.	Removal of native valve
A5.	Valve-replacement
**Points (max. 5):**	
**B.**	**Likelihood of success**
B1.	50% chance of prolonging the patient’s life
**Points (max. 1):**	
**C.**	**Procedural steps post-operatively**
C1.	Insertion of suction drainage
C2.	Step-down care (intensive, normal, rehab)
**Points (max. 2):**	
**D.**	**General main complications**
D1.	Superficial infection of the incision
D2.	Bleeding
D3.	Cutaneous nerve damage
D4.	Pain
D5.	Wound healing defects
**Points (max. 5):**	
**E.**	**Specific main complications**
E1.	Pneumothorax
E2.	Heart rhythm disturbances
E3.	Myocardial infarction
E4.	Mediastinitis
E4.	Endocarditis
E5.	Pericardial effusion/tamponade
E6.	Ischemic stroke
**Points (max. 7):**	
**TOTAL POINTS (max. 20):**	

Example of a checklist (severe aortic valve defect) with a standardized set of 20 items of information.

### Interventions

The intervention comprised a 30-minute communication training, in which the medical students were introduced to several strategies and techniques, based on Brown’s framework for collaborative decision-making (BFCDM), Paterick’s ideal statute (PIS) and Baile’s SPIKES-protocol (BSP) [21-23]. The lecture comprised the following: a role play with an exceptionally bad example of an informed consent talk, then an interactive brainstorming session on what contributes to and hinders a medical laypersons’ comprehension, a short lecture on the theory, and a role play with an appropriate informed consent talk. Main aspects of the theoretical input included

i) Setting the conditions in advance, e.g. sitting position (BSP),

ii) Assessing what the patient already knows and how much additional information is wanted followed by the description of the procedure, its risks and possible alternatives (BSP, BFCDM, PIS),

iii) Using easy and understandable language adapted to the patient’s level (BSP),

iv) Active encouragement to ask questions (BSP, BFCDM),

v) Making use of the available information sheets for medical procedures.

vi) Reducing the amount of information by clustering the facts (BSP). For example, combining each operative step with its possible complication.

At the end of each thematic teaching subunit, students applied cognitive load reduction techniques in preparation for their next ICT. They drafted elements of the informed consent talks and received feedback on their performance from fellow students and the instructors.

The comparison intervention was a 30-minute interactive class on ECG interpretation. No communication training was given in order to control for the Hawthorne effect and to blind the medical laypersons.

### Assessment

After the simulated informed consent talks, the medical laypersons were asked to record on a blank sheet of paper, what they recalled from the simulated informed consent talks. The same strategy was used by Fortun et al. in their study on information recall, but since we standardized our ICTs with only 20 information items in contrast to the 13-page information sheet in Fortun’s study, no prompts were given [5].

### Statistics

We used JMP 9.0 (SAS Institute Inc, Cary, NC, USA), nQuery 7.0 (Statistical Solutions, Saugus, MA, U.S.A.) and SPSS 19.0 (SPSS Inc., Chicago, IL, USA) to compute the results. Calculations were based on the mean of the raters’ evaluations. We compared the results of the recall-tests using Student’s *t*-test for matched pairs. The interrater-reliability was calculated as proposed by Shrout and Fleiss [24]. An improvement of 15% in number of remembered information items (three or more) was considered to be clinically significant.

### Ethics

The study protocol was approved by the local ethics committee, decision number 488/2010A. The study was conducted in accordance to the declaration of Helsinki, revised form, Seoul 2008. All participants (medical laypersons and medical students) gave written informed consent. Study participation was voluntary. Both medical students and medical laypersons received a small financial compensation.

## Results

One set of data had to be excluded due to missing data since the medical layperson only recorded, whether she enjoyed the talk, not what she remembered. Two experienced physicians acted as raters, the interrater-reliability was 0.8.

The characteristics of the medical students and medical laypersons are shown in tables 2 and 3. Previous ICT experience had no measurable effect on the information recall of the medical laypersons.

**Table 2:** Characteristics of medical students

	**Early intervention group (EI)**	**Late intervention group (LI)**	**p**
Age	26 ± 6 years	24 ± 2 years	.11
Mean + SD			
Gender	4 male,	8 male,	.10
	10 female	7 female	
Previous training	Paramedic (n = 1)	Nurse (n = 2)	.43
	Economics (n = 1)		
Clinical experience	12 5th year	15 5thyear	.01
	2 6th year		
Mean + SD	17 ± 3 weeks of clerkships	15 ± 5 weeks of clerkships	
ICTs attended			.29
Mean + SD	6.6 ± 9.4	12.8 ± 24.8	
ICTs conducted			.48
Mean + SD	1.1 ± 1.7	1.3 ± 2.0	

Characteristics of the medical students. Early intervention group: the medical laypersons received simulated informed consent talks by medical students, who had a communication training first and a comparison intervention after. Late intervention group: the medical laypersons received simulated informed consent talks by medical students, who had a comparison intervention first and a communication training after. The medical laypersons were blinded to the interventions of the medical students. “ICTs attended” and “ICTs conducted” refer to the specific experience of the medical students concerning ICTs. The p-values were calculated using the Chi Square test.

**Table 3:** Characteristics of the medical laypersons

	**Early intervention group (EI)**	**Late intervention group (LI)**	**p**
Gender	9 female, 6 male	8 female, 7 male	.53
Education	4 academic, 11 non-academic	6 academic, 9 non-academic	.16
Age + SD	46 ± 10 years	49 ± 7 years	.18

Characteristics of the medical laypersons. Early intervention group: the medical laypersons received simulated informed consent talks by medical students, who had a communication training first and a comparison intervention after. Late intervention group: the medical laypersons received simulated informed consent talks by medical students, who had a comparison intervention first and a communication training after. The p-values were calculated using the Chi Square test.

**Figure 2:**
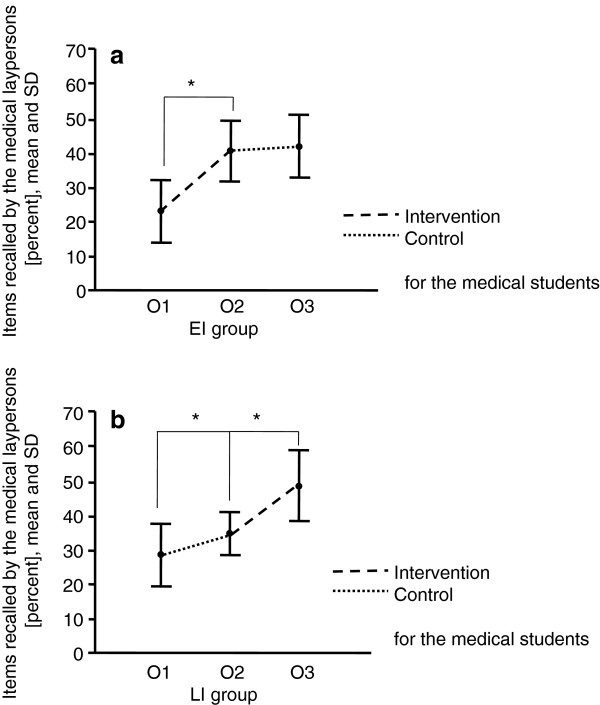
**Items recalled by the medical laypersons after a simulated informed consent talk with an advanced medical student before and after a communication training that taught the students to reduce the layperson’s cognitive load and a comparison intervention on ECG interpretation, respectively. a**: EI: Early intervention group, the medical students received first the communication training, then the comparison intervention. **b**: LI: late intervention group: the medical students received first the comparison intervention, then the communication training.

In both groups the medical laypersons recalled significantly more items after the medical students received the communication training (EI group: 41 ± 9% vs. 23 ± 9%, p < .0001, LI group 49 ± 10% vs. 35 ± 6%, p < .0001). After the comparison intervention the gain in recalled information items was modest in both the EI and LI groups, and it was significant only in the LI group (EI group: 42 ± 9% vs. 40 ± 9%, p = .41, LI group 35 ± 6% vs. 29 ± 9%, p = .016) (see Figure 2).

## Discussion

In our study a short communication training for advanced medical students focusing on strategies that reduce cognitive load significantly improved recall of information by medical laypersons after a simulated informed consent talk (ICT).

While the investigations of previous studies on informed consent talks concentrated mostly on factors regarding the patients, our study implicates that there is potential in improving the efficacy of informed consent talks by using interventions for the physicians conducting ICTs.

Other studies focusing on optimizing the ICTs used much longer communication teachings lasting 2–5 days to improve their participants’ abilities [25,26]. Flory and colleagues concluded that besides using a standard process for informed consent, the personal contact with a qualified staff seems to be the best approach to secure as much comprehension by the patients as possible [13]. The fact that a very short intervention had a measurable effect in our study could be attributed to the fact that we approached medical students, who up to this point have not really practiced ICTs so that they were still very susceptible to even little teaching input. After the comparison intervention there was a small improvement in the late intervention group. This could be attributed to an unspecific training effect of either the medical students or the medical laypersons, since the medical laypersons listened to three ICTs. It is likely that practice, even without specific training for medical students/physicians conducting the ICTs, improves the efficacy of the ICTs. In our study, we asked the medical students about their specific experience with ICTs but could not find an effect on the recall of information items in the medical laypersons. Our study was not designed to show this effect, so this question should be addressed by a differently designed study.

There are several limitations to our study. We used laboratory conditions in order to standardize possible confounders and the intrinsic cognitive load. Thus, the medical laypersons in our study were somewhat younger than typical patients suffering from the diseases. They were not in distress and not emotionally involved. It is well-known that emotional factors have a serious influence on the perception of situations and the actual emotional load of a real situation might reduce the amount of remembered details [27]. However, one would expect that fear and emotional distress affected mainly germane cognitive load, so that optimizing the extrinsic and intrinsic cognitive loads is of even more importance. Although the communication training aimed at teaching medical students how to reduce a cognitive load, we did not measure layperson’s cognitive load, but the “hard outcome”, the number of information items recalled after the simulated informed consent talks. Furthermore, despite the standardized setting of this study, we cannot exclude confounders. There is the possibility of a selection bias. We actively encouraged all regular students to take part in our study. Nonetheless, we cannot rule out that only students who were aware of their own limitations and consequently were more susceptible to even short training sessions agreed to participate in our study. On the other hand, it is equally possible that only very motivated students participated, thus their performance was above average. The students were not blinded to the intervention, the comparison-intervention served as blinding for the medical laypersons. In addition, the novelty effect of cases could play a role in performance. By familiarizing the students with the cases prior to the actual study, we tried to reduce the possibility of this effect. Finally, although two expert reviewers considered the used cases equally relevant and difficult, an (un-)equivalence of the cases used cannot be excluded completely. In order to control for this bias, we arranged them in a Latin-square design (C_heart_–C_kidney_–C_pancreas_,C_pancreas_–C_heart_–C_kidney_, C_kidney_–C_pancreas_–C_heart_) so that each case was equally distributed in position. Finally, we can’t exclude the possibility that the medical laypersons looked up the content of the planned operations before the study, even though they all signed an agreement not to.

Despite these possible restrictions we conclude that a specific training in conducting ICTs for advanced medical students improves the recall of information of medical laypersons and thus may enhance patient safety as more informed patients are better able to actively participate in the treatment process. Further studies need to address the transferability into daily clinical routine with doctors and actual patients to see how the findings hold up in real life situations.

## Conclusion

Communication training for advanced medical students can improve the information recall of medical laypersons in simulated informed consent talks.

## Competing interests

The authors declare that they have no competing interests.

## Authors’ contributors

AW and NC were responsible for the study design, data analysis and drafting the manuscript. FH and SZ contributed significantly to the study design and critically revised the manuscript. NS performed the statistical analysis and critically revised the manuscript. PW and RR instructed the students and critically revised the manuscript. All authors approved the final manuscript.

## Pre-publication history

The pre-publication history for this paper can be accessed here:

http://www.biomedcentral.com/1472-6920/13/15/prepub
